# Attitudes and awareness of medical assistance while traveling abroad

**DOI:** 10.1186/s12992-018-0382-5

**Published:** 2018-07-11

**Authors:** Yi-Hsuan Lee, Chia-Wen Lu, Pei-Zu Wu, Hsien-Liang Huang, Yi-Chun Wu, Kuo-Chin Huang

**Affiliations:** 1 Department of Family Medicine, National Taiwan University Hospital, No.7, Zhongshan S. Rd., Zhongzheng District, Taipei, Taiwan; 2 Department of Family Medicine, National Taiwan University Hospital Bei-Hu Branch, No.87, Neijiang St., Wanhua District, Taipei, Taiwan; 3 Community and Geriatric Research Center, National Taiwan University Hospital, Bei-Hu Branch, No.87, Neijiang St., Wanhua District, Taipei, Taiwan; 4 Department of Family Medicine, Taipei City Hospital Heping Fuyou Branch, No.33, Sec. 2, Zhonghua Rd., Wanhua District, Taipei, Taiwan; 5 Centers for Disease Control, No.6, Linsen S. Rd., Zhongzheng District, Taipei, Taiwan

**Keywords:** Travel medicine, Medical assistance, Emergency medical services, Insurance, health, Health attitudes

## Abstract

**Background:**

With globalization, more and more people travel to countries where they are at risk of injuries and travel-related diseases. To protect travelers’ health, it is crucial to understand whether travelers accurately perceive medical assistance resources before and during their trips. This study investigated the need, awareness, and previous usage of overseas emergency medical assistance services (EMAS) among people traveling abroad.

**Methods:**

Anonymous questionnaires were distributed to patients (*n* = 500) at a travel clinic in Taipei, Taiwan.

**Results:**

The results showed that EMAS were important, especially in the following categories: 24-h telephone medical consultation (91.8%), emergent medical repatriation (87.6%), and assistance with arranging hospital admission (87.4%). Patients were less aware of the following services: arrangement of appointments with doctors (70.7%) and monitoring of medical conditions during hospitalization (73.0%). Less than 5% of respondents had a previous experience with EMAS.

**Conclusions:**

EMAS are considered important to people who are traveling abroad. However, approximately 20–30% of travelers lack an awareness of EMAS**,** and the percentage of travelers who have previously received medical assistance through these services is extremely low. The discrepancy between the need and usage of EMAS emphasizes the necessity to adapt EMAS materials in pre-travel consultations to meet the needs of international travelers.

**Electronic supplementary material:**

The online version of this article (10.1186/s12992-018-0382-5) contains supplementary material, which is available to authorized users.

## Background

The number of international travelers is increasing each year. In 2016, international tourist arrivals in all countries reached a total of 1235 million, representing a 3.9% increase over the prior year [1]. This number has been increasing for seven consecutive years and is projected to increase to approximately 1.8 billion international tourists by 2030 [1, 2]. Among these travelers, travelers visiting Asia and the Pacific region led this growth by an 8% increase in international tourism in 2016 [1].

People who travel abroad have a greater risk of morbidities or death. Travelers to developing countries have twice the risk of injuries from traffic accidents than their counterparts in their home countries [3]. Approximately 20 to 25% of traveler deaths are caused by injuries, which are mostly road traffic injuries [4]. Depending on the destination, approximately 22 to 64% of international travelers have some illness during their trip, but most of these illnesses are mild and self-limited [5]. However, it is estimated that approximately 8% of travelers reporting travel illness require medical attention, with 0.3% requiring hospital admission either during their trip or upon their return from a developing country [6].Although it is difficult to determine the actual risk to international travelers by destination from the limited amount of information available, Asia and Sub-Saharan Africa were the most common regions in which travel-related health problems occurred according to recent data from the GeoSentinel network, a global provider-based network of travel and tropical medicine clinics [7].

In an era of extremely high medical expenses for emergency medical care, severe illness or injuries may result in financial catastrophe or impoverishment for international travelers. Considering the increased risk of injuries and illness during international trips, travel insurance is critical for travelers with medical needs during travel [8, 9]. Travel insurance provides coverage for outpatient, emergency, and inpatient medical expenses that occur during trips, which are not typically covered by other health insurance plans [8]. In addition, 24-h instant medical consultation assistance is provided by agents contracted by the insurance company. Travel insurance is especially important for travelers from low- or lower-middle-income countries, where incomes and average medical care payments are much lower than those of upper-**,** middle- or high-income countries. Furthermore, aeromedical evacuation (AME), which can cost between $25,000- $250,000, is not affordable for most travelers without travel insurance [10]. Overseas emergency assistance services (EMAS) are routinely included when travelers purchase travel medical insurance.

Due to the expansion and heightening of international tourism, the frequencies of travel injuries and illnesses have been increasing every year. In an Australian study, 25.3% of insured travelers reported the use of emergency assistance during travel [11]. However, there is a lack of studies that explore the need and awareness of medical assistance while traveling abroad. Hence, this study aims to investigate the knowledge, attitudes, and awareness of EMAS of travelers while they are traveling abroad and increase the awareness of EMAS in primary care providers and public health officials.

## Methods

### Design

This study was a cross-sectional survey using a well-structured, three-part questionnaire that was distributed to all patients at the travel clinic in National Taiwan University Hospital (NTUH) from April 2014 to May 2014. Return and completion of the questionnaire represented the subject’s consent of participation. The questionnaire was completed anonymously.

### Subjects

The targeted participants were all adult patients over 18 years old with prior experience traveling abroad. Patients who were unwilling or unable to complete the questionnaire were excluded. The design of the study and selection of subjects were approved by the Research Ethics Committee at National Taiwan University Hospital in Taiwan (201402078RINB) before the study was conducted.

### Questionnaire

The three-part questionnaire included questions on socio-demographic characteristics, attitudes, and awareness towards the EMAS commonly provided to insured travelers. The questionnaire was pretested for face validity by a panel of ten physicians with experience in the clinical practice of travel medicine, including family medicine specialists, infectious disease specialists and pediatricians, from NTUH and Centers for Disease Control (CDC), Taiwan. A literature review and consensus opinion from five physicians at NTUH and CDC, Taiwan, were also conducted to test the content validity of the questionnaire. However, few studies have investigated the perceived importance and awareness of preparedness measures during travel, such as EMAS provided by insurance companies in this study. To the best of our knowledge, other measures are not available to correlate our results with. The five physicians were asked to verify whether the factors regarding travel risk assessments in part I of the questionnaire were appropriate and whether the EMAS content listed in parts II and III of the questionnaire were services that they considered important or that travelers mostly mentioned in clinical practice. Each item was evaluated on a scale of one (low) to five (high) for clarity and relevance to clinical practice, and items with a rating of at least four were included. After the process, we added the service of “medical consultation via instant messaging” and deleted “the transportation and transfer of medical supplies” in parts II and III of the questionnaire. We also used reliability analysis to test for internal consistency. Cronbach’s alpha was 0.907 for items of importance and 0.945 for items of awareness and usage experience (see Additional file 1).

The included socio-demographic characteristics were sex, age, education, medical history, trip destination, special activities during travel, and prior travel-related health problems. The other two parts of the questionnaire included the following components:

- Attitude towards EMAS: This part examined the subjects’ perceptions regarding the importance of nine different features of EMAS during travel as follows: (1) 24-h telephone medical advice, (2) instant messaging of medical advice, (3) arrangement of appointments at nearby hospitals, (4) arrangement of appointments with doctors, (5) medical record translation and transfer, (6) arrangement of hospital admission, (7) monitoring of medical conditions during hospitalization, (8) emergency medical repatriation, and (9) arrangement of appointments with doctors after travel. The scoring system used a five-point Likert Scale, ranging from “very unimportant” (1 point) to “unimportant” (2 points), “no comment” (3 points), “important” (4 points) and “very important” (5 points). Higher scores indicated positive attitudes regarding the need of certain services. Services with a score of 1 to 3 points were considered “not important” and those with a score of 4 to 5 points were considered “important.”
- Awareness and previous experience of EMAS: This part sought information on whether people had heard about the abovementioned nine different features of travel insurance services and whether they had experience using these services in the past.

### Statistical analysis

Data management and statistical analysis were performed using SPSS17.0 statistical software (SPSS, Chicago, IL). Demographic data were represented by frequency distributions. Chi-square test was used to compare the proportions of the importance and awareness of EMAS between different socio-economic variables. A *P* value less than 0.05 was considered statistically significant. Missing data were excluded from analysis.

## Results

A total of 615 patients who visited the travel clinic were given the questionnaire and 501 responded (effective response rate = 81.3%). The unbiased selection and high response rate represented good interval validity. After eliminating one incomplete questionnaire, 500 respondents were included in the final analysis (208 males and 292 females).

### Demography

Table 1 shows the demographic characteristics of the survey respondents. The mean age of the respondents was 29.92 ± 11.08 years. 59.2% of the respondents had a university or college degree, whereas 34% had graduate or higher degrees. Thirteen percent of respondents reported a medical history of chronic illness**,** and 14.2% had previously experienced travel-associated illness while abroad. Furthermore, 45.8% of the respondents planned to travel to a developing region, such as China, Southeast and South Asia, Middle and South America, or Africa**, **at the time of their visit. A total of 16.6% the respondents planned to participate in activities that may increase their risk of health problems, such as mountain climbing or jungle trekking, during their travel.

**Table 1:** Demographic characteristics of survey respondents (*N* = 500)

Characteristic	Number	Percentage (%)
Age(29.92 ± 11.08 years)		
18–29	319	63.9
30–39	105	21.0
40–49	36	7.2
50–69	39	7.8
Sex		
Male	208	41.6
Female	292	58.4
Highest educational level completed		
High School or below	34	6.8
University or College	296	59.2
Graduate School and higher	170	34.0
Medical History^a^		
Yes	65	13.0
No	435	87.0
Planned travel destinations		
China	34	6.8
North Asia	36	7.2
Southeast and South Asia	67	13.4
North America	212	42.4
Middle and South America	44	8.8
Europe and Oceania	19	3.4
Africa	50	10.0
Multiple destinations	34	6.8
Travel-associated illness^b^		
Yes	71	14.2
No	429	85.6
Planned special activities during travel^c^		
Yes	83	16.6
No	417	83.4

^a^hypertension, diabetes, cardiovascular diseases, arrhythmia, asthma, gout, cancer, anemia

^b^fever, common cold, traveler’s diarrhea, cellulitis, urinary tract infection, chicken pox, malaria, typhoid fever, toothache, acute mountain sickness, accidental injury, sprain

^c^mountain climbing, scuba diving, river rafting, snow skiing, surfing, jungle trekking, pilgrimage

### Importance, awareness, and previous experience of EMAS

**Fig. 1:**
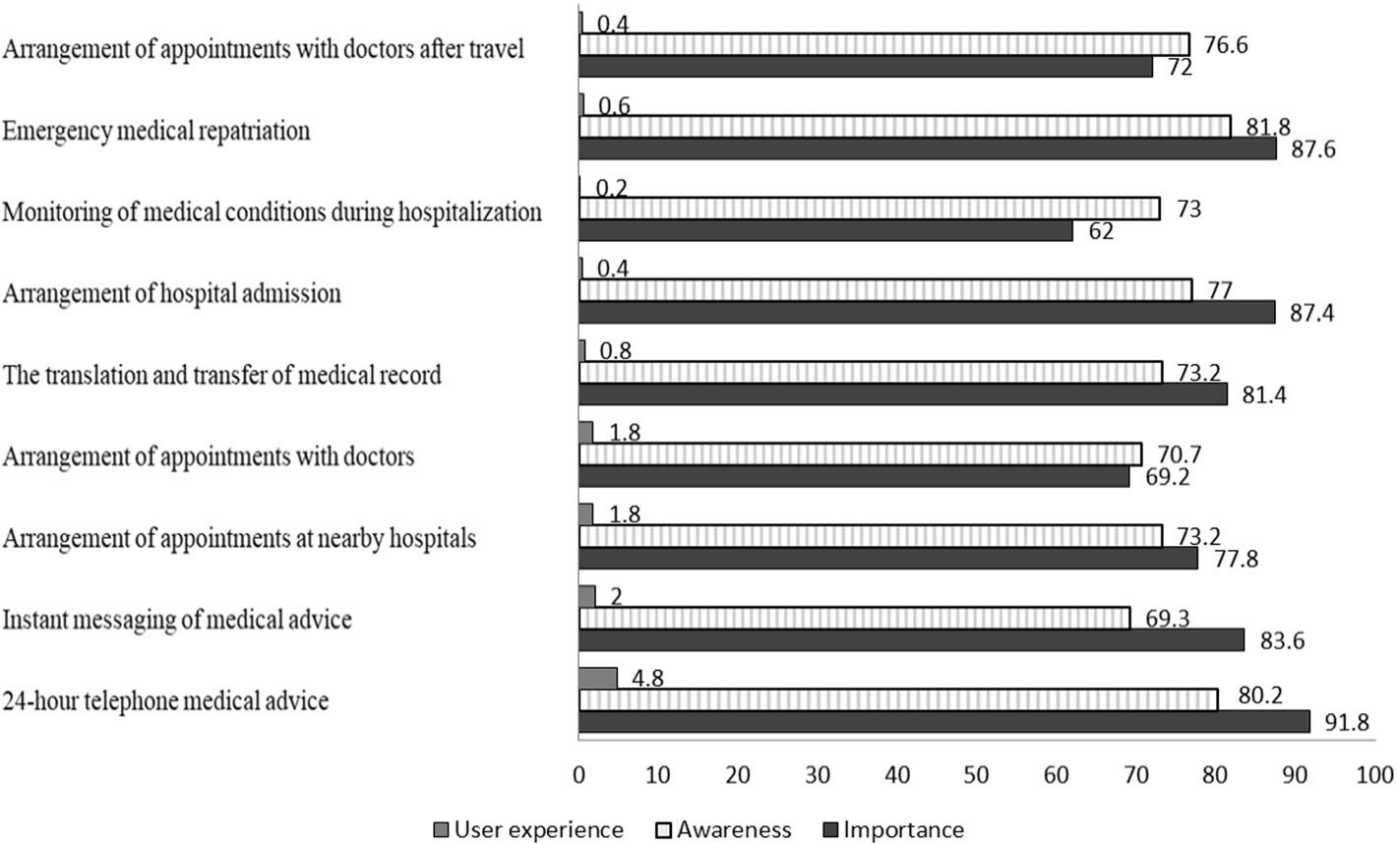
Percentage of travelers’ ratings of importance, awareness, and experience with previous usage of emergency medical assistance services

Figure 1 shows the respondents’ ratings of the importance, awareness, and previous experience of each emergency medical assistance service. The top three most highly rated EMAS include 24-h telephone medical consultation (91.8%), emergent medical repatriation (87.6%), and providing a referral for hospital admission of tourists abroad (87.4%). The services that the subjects were least aware of included arranging appointments with doctors (70.7%), monitoring medical conditions during hospitalization (73.0%), and the translation and transfer of medical records (73.2%). Less than 5% of the respondents had previously experienced any EMAS.

### Importance of EMAS and demographic characteristics

Table 2 shows the association between the importance of EMAS and demographic characteristics for the top three most highly rated services. A significant association was observed between the importance of emergency medical repatriation with age, sex, and respondents with previous travel-associated illness. The importance of arranging for hospital admission was associated with age. Subjects who were middle aged (30–49 years old), were male, and had previously experienced travel-associated illness were more likely to consider these services to be less important.

**Table 2:** Association between demographic characteristics and the three most important emergency medical services as considered by travelers (N = 500)

		24-h telephone medical consultation		Emergent medical repatriation		Arrangement of hospital admission	
Variable	Number	Importance (%)	*P* Value*	Importance (%)	*P* Value*	Importance (%)	*P* Value*
Age(years)			0.917		0.044^+^		0.001^+^
18–29	319	92.2		90.0		91.2	
30–39	105	92.4		86.7		81.0	
40–49	36	88.9		75.0		72.2	
50–69	39	92.3		82.1		87.2	
Sex			0.497		0.024^+^		0.113
Male	208	92.8		83.7		84.6	
Female	292	91.1		90.4		89.4	
Highest educational level completed			0.271		0.288		0.263
High School or below	34	85.3		85.3		91.2	
University or College	296	91.6		89.5		88.9	
Graduate School and higher	170	93.5		84.7		84.1	
Medical History			0.075		0.112		0.468
Yes	65	86.2		81.5		84.6	
No	435	92.6		88.5		87.8	
Planned travel destinations			0.874		0.550		0.117
China	34	91.2		88.2		85.3	
North Asia	36	94.4		88.9		88.9	
Southeast and South Asia	67	91.0		92.5		88.1	
North America	212	91.5		89.2		91.0	
Middle and South America	44	93.2		81.8		81.8	
Europe and Oceania	19	84.2		78.9		89.5	
Africa	50	90.0		82.0		74.0	
Multiple destinations	34	97.1		85.3		88.2	
Travel-associated illness			0.188		0.024^+^		0.983
Yes	71	91.1		86.2		87.3	
No	429	95.8		95.8		87.4	
Planned special activities during travel			0.095		0.054		0.868
Yes	83	96.4		94.0		88.0	
No	417	90.9		86.3		87.3	

*The *p* Value was calculated using the chi-square test for the analysis

^+^Items with *p* Value < 0.05

### Awareness of EMAS and demographic characteristics

Table 3 shows the association between the awareness of EMAS and demographic characteristics in the three services that the subjects were least aware of. There were no significant differences between awareness of the three services and demographic characteristics.

**Table 3:** Association between demographic characteristics and the three emergency medical services that travelers are least aware of (*N* = 500)

		Arrangement of appointments with doctors		Monitoring of medical conditions during hospitalization		The translation and transfer of medical record	
Variable	Number	Awareness (%)	*P* Value*	Awareness (%)	*P* Value*	Awareness (%)	*P* Value*
Age(years)			0.696		0.757		0.954
18–29	319	68.9		71.5		72.7	
30–39	105	73.3		74.3		73.3	
40–49	36	75.0		77.8		72.2	
50–69	39	74.4		76.9		76.9	
Sex			0.755		0.323		0.879
Male	208	71.5		70.7		73.6	
Female	292	70.2		74.7		72.9	
Highest educational level completed			0.154		0.108		0.590
High School or below	34	81.8		88.2		79.4	
University or College	296	67.9		71.3		73.6	
Graduate School and higher	170	73.5		72.9		71.2	
Medical History			0.555		0.642		0.670
Yes	65	73.8		75.4		75.4	
No	435	70.3		32.6		72.9	
Planned travel destinations			0.833		0.741		0.964
China	34	76.5		79.4		76.5	
North Asia	36	80.6		75.0		77.8	
Southeast and South Asia	67	68.7		67.2		70.1	
North America	212	69.7		73.1		72.6	
Middle and South America	44	70.5		75.0		75.0	
Europe and Oceania	19	78.9		84.2		78.9	
Africa	50	68.0		68.0		72.0	
Multiple destinations	34	67.6		76.5		73.5	
Travel-associated illness			0.363		0.414		0.108
Yes	71	66.2		69.0		67.6	
No	429	71.5		73.7		74.1	
Planned special activities during travel			0.163		0.083		0.090
Yes	83	77.1		80.7		80.7	
No	417	69.5		71.5		71.7	

*The *p* Value was calculated using the chi-square test for the analysis

## Discussion

Among the respondents in this study, 14.2% of subjects reported previous travel-associated illness while traveling. In other studies, approximately 20–60% of travelers experienced health problems during travel [5, 12], which is much higher than the rate found in our study. The reason for this difference might be due to the younger age of our respondents, 84.9% of our subjects were 18 to 39 years old.

In our study, the services that our respondents found to be important include 24-h telephone medical consultation (91.8%), emergent medical repatriation (87.6%), and providing a referral for hospital admission to tourists abroad (87.4%). In an Australian study, emergency telephone service provided by the travel insurance company was reported in 17.1% of all general claims [13]. The percentage of respondents who previously experienced EMAS was lower than 5% in our study, which might have resulted from the low awareness or reduced need of these services by respondents with minor illness while traveling. This percentage is a small number, in comparison to an Australian study, where after an Asian tsunami, 40.9% of travelers submitting tsunami-related claims to their insurance company received emergency medical assistance and consultation [14]. Services to help arrange hospital admission and emergency medical repatriation were considered less important in middle-aged respondents in our study. This response is possibly because middle-aged travelers are less vulnerable to illness and injury and are more resourceful when encountering health emergencies than teenagers or the elderly. Respondents with previous experiences of travel-associated illness did not consider emergency medical repatriation to be more important. The underlying causal relationship warrants further study.

This study demonstrated that emergent medical repatriation was considered one of the most important EMAS by travelers. Emergent medical repatriation is an interprofessional collaborative practice, which includes the field of emergency medicine and travel medicine [15]. Travelers are encouraged to apply for travel insurance to cover the possible health hazards that may arise during travel, especially when medical evacuation and repatriation are not covered by general health insurance. Travelers should also be advised as to how to access emergency medical assistance while abroad, which has been discussed in an Australian study [16]. The EMAS provided by insurance companies can generally reduce the economic burden of travel-associated illnesses and offer information on and aid with medical services abroad for travelers [8, 17]. Many of our respondents had a risk of health-related issues during travel: 13% had pre-existing diseases, 16.6% were planning on participating in risky activities, and 45.8% were traveling to a developing country. Although these subjects were highly educated, with 93.2% having an education level equal to or higher than university or college, 19.8 to 29.3% of these respondents were not aware of the services provided by EMAS, which gives them an increased risk of inadequate medical care while traveling abroad.

Our results showed that many travelers were not aware of the service that provides medical consultation through instant messaging because this service is not routinely offered by insurance companies in Taiwan. The pre-travel consultation rate is relatively low in Taiwan at 0.1% compared to the 40.5% rate for all ill GeoSentinel travelers [7]. Another survey showed that 52.1% of European travelers who were going to visit a developing country sought pre-travel consultations [18]. Travelers who did not visit a travel clinic may have a higher need of EMAS and less awareness of these services due to their lower education level.

Prior travel experience was an inclusion criterion in this study. Regarding the perceived importance and awareness of EMAS, we believe that compared to first-time travelers, more experienced travelers would be more aware of EMAS and would consider EMAS to be more important because of possible previous travel-associated illness experiences. However, our results demonstrated that even experienced travelers may lack an awareness of EMAS during travel. Therefore, there is a need for primary care providers and public health officials to incorporate travel medical assistance materials in pre-travel consultation and refine travel clinic practices and health policies.

### Limitations

There are several limitations in this study. First, 81.3% of the surveyed patients returned a completed questionnaire. A crowded clinic setting might have influenced the effective response rate and might have resulted in selection bias. Second, due to a lack of collected data regarding the previous travel experience of the respondents, we did not adjust the outcomes in this study. This work was a pilot study on EMAS, and we would like to further clarify the risk level of the travelers’ previous trips in a subsequent study. Third, since most of the respondents at the travel clinic were young, had high education levels, and had fewer pre-existing diseases and travel-associated illness experiences, some of the results of this study might not apply to all travelers. Lastly, because most of the travelers at the travel clinic in NTUH came from northern Taiwan, the respondents in this study may not be representative of travelers from all areas of Taiwan. However, the travel clinic in NTUH serves approximately 30–59% of all travelers visiting travel clinics. A total of 5,914 travelers visited the travel clinic in NTUH in 2017, whereas approximately 10,000- 20,000 travelers visited travel clinics in Taiwan over a year [19]**.** The respondents in this study could be representative of the majority of the travelers visiting travel clinics in Taiwan.

Despite the above limitations, few studies have investigated the need, awareness, and experience of EMAS while traveling abroad. The questionnaire in the study was developed for this work and has not been used before. We believe that the perceived importance, awareness and usage experiences of EMAS are good preliminary indicators for exploring whether the availability of EMAS or other medical assistance services during travel is indeed an issue. This investigation revealed that approximately 20–30% of travelers lacked an awareness of EMAS during travel despite their overall positive attitude towards the importance of these services. In a European study, most travelers sought advice from their primary care physician for pre-travel consultation and about one-third of travelers visited a travel clinic specialist [18]. Previous studies have also shown that approximately 60% of primary care physicians in New Zealand [20] and 39% of travel clinics worldwide [21] regularly advise travelers about travel insurance.

## Conclusions

The cross-sectional survey in a travel clinic clearly demonstrates that EMAS is necessary for international travelers. EMAS are considered important for most international travelers. However, approximately 20–30% of the travelers lacked an awareness of EMAS, and the percentage of travelers who had previously received medical assistance through these services is extremely low. Primary care physicians and travel medicine specialists are encouraged to offer more information about emergency medical assistance resources during pre-travel consultation, empowering travelers to manage their health throughout the trip.

## Additional file

Additional file 1: Questionnaire: Assessment of Travel Risks and Needs for Travel Medical Assistance of Patients at Travel Clinics. (DOCX 21 kb)
