# Supplementary material for: Attitudes and awareness of medical assistance while traveling abroad
Source: Global Health. 2018 Jul 11;14:67. doi: 10.1186/s12992-018-0382-5 (PMC6042459; doi:10.1186/s12992-018-0382-5)
Supplement: Supplementary file 1 — Questionnaire: Assessment of Travel Risks and Needs for Travel Medical Assistance of Patients at Travel Clinics. (DOCX 21 kb) [file 12992_2018_382_MOESM1_ESM.docx]

**Assessment of Travel Risks and Needs for Travel Medical Assistance of Patients at Travel Clinics**

The purpose of this study is to examine people’s needs for pre-travel consultation and travel medical assistance in order to refine travel clinic practices and health policies. The study is being conducted through National Taiwan University Hospital and Centers for Disease Control (Taiwan). Your responses will be anonymous and will never be linked to you personally. Return and completion of the questionnaire represented your consent of participation of the study. Thank you for your cooperation.

**Part 1: Personal information and travel risk assessments**

1. Age(in years)： ______
2. Sex： □Male　□Female
3. The highest level of education you have completed： □no formal education □elementary school □junior high school □high school or equivalent □Bachelor’s degree □Master’s degree, Doctoral degree or Professional degree
4. Medical history：□No □Hypertension □Diabetes □Heart Disease □Asthma □Acute mountain sickness □Cancer_______ □Surgery____________ □Others____________□Not Sure
5. Travel destinations of this trip (Multiple): □Mainland China □Hong Kong or Macau □Japan or Korea □Southeast Asia □Southern Asia □North America □Central and South America □Europe □New Zealand or Australia □Africa □Others____________
6. Planned special activities for this trip：□None □Yes：□Scuba diving □Mountain climbing □River rafting □Others______
7. Travel-associated illness before:

□No □Yes：□Common Cold □Acute gastroenteritis □Acute mountain sickness □Sprains □Accidents □Others____________；Need for medical assistance due to travel-associated illness：□No □Yes

**Part 2 and 3: Need Assessment for Travel Medical Assistance**

| Need Assessment for Travel Medical Assistance | Importance | | | | | Previous usage | | |
| --- | --- | --- | --- | --- | --- | --- | --- | --- |
|  | 1. Very Unimportant 2. Unimportant 3. No Comment 4. Important 5. Very Important | | | | | 1. Never heard before 2. Not used 3. Used | | |
|  | 5 | 4 | 3 | 2 | 1 | 3 | 2 | 1 |
| 1. 24-hour telephone medical **advice** | □ | □ | □ | □ | □ | □ | □ | □ |
| 1. **Instant messaging of medical advice** | □ | □ | □ | □ | □ | □ | □ | □ |
| 1. Arrangement **of** appointments at nearby hospitals | □ | □ | □ | □ | □ | □ | □ | □ |
| 1. Arrangement **of** appointments with doctors | □ | □ | □ | □ | □ | □ | □ | □ |
| 1. The translation and transfer of medical **record** | □ | □ | □ | □ | □ | □ | □ | □ |
| 1. Arrangement **of** hospital admission | □ | □ | □ | □ | □ | □ | □ | □ |
| 1. Monitoring of medical **conditions** during hospitalization | □ | □ | □ | □ | □ | □ | □ | □ |
| 1. Emergent medical repatriation | □ | □ | □ | □ | □ | □ | □ | □ |
| 1. Arrangement **of** appointments with doctors after travel | □ | □ | □ | □ | □ | □ | □ | □ |
| 1. Other Services you think important in travel medical assistance? | Please describe: | | | | | | | |
